# Protective Role of HLA-DRB1*13:*02* against Microscopic Polyangiitis and MPO-ANCA-Positive Vasculitides in a Japanese Population: A Case-Control Study

**DOI:** 10.1371/journal.pone.0154393

**Published:** 2016-05-11

**Authors:** Aya Kawasaki, Narumi Hasebe, Misaki Hidaka, Fumio Hirano, Ken-ei Sada, Shigeto Kobayashi, Hidehiro Yamada, Hiroshi Furukawa, Kunihiro Yamagata, Takayuki Sumida, Nobuyuki Miyasaka, Shigeto Tohma, Shoichi Ozaki, Seiichi Matsuo, Hiroshi Hashimoto, Hirofumi Makino, Yoshihiro Arimura, Masayoshi Harigai, Naoyuki Tsuchiya

**Affiliations:** 1 Molecular and Genetic Epidemiology Laboratory, Faculty of Medicine, University of Tsukuba, Tsukuba, Japan; 2 Doctoral Program in Biomedical Sciences, Graduate School of Comprehensive Human Sciences, University of Tsukuba, Tsukuba, Japan; 3 Master’s Program in Medical Sciences, Graduate School of Comprehensive Human Sciences, University of Tsukuba, Tsukuba, Japan; 4 Department of Pharmacovigilance, Graduate School of Medical and Dental Sciences, Tokyo Medical and Dental University, Tokyo, Japan; 5 Department of Rheumatology, Graduate School of Medical and Dental Sciences, Tokyo Medical and Dental University, Tokyo, Japan; 6 Department of Nephrology, Rheumatology, Endocrinology and Metabolism, Okayama University Graduate School of Medicine, Dentistry and Pharmaceutical Sciences, Okayama, Japan; 7 Department of Internal Medicine, Juntendo University Koshigaya Hospital, Koshigaya, Japan; 8 Department of Internal Medicine, St. Marianna University School of Medicine, Kawasaki, Japan; 9 Clinical Research Center for Allergy and Rheumatology, Sagamihara Hospital, National Hospital Organization, Sagamihara, Japan; 10 Department of Nephrology, Faculty of Medicine, University of Tsukuba, Tsukuba, Japan; 11 Department of Internal Medicine (Rheumatology), Faculty of Medicine, University of Tsukuba, Tsukuba, Japan; 12 Department of Nephrology, Nagoya University Graduate School of Medicine, Nagoya, Japan; 13 Juntendo University School of Medicine, Tokyo, Japan; 14 Okayama University Hospital, Okayama, Japan; 15 First Department of Internal Medicine, Kyorin University School of Medicine, Tokyo, Japan; 16 Institute of Rheumatology, Tokyo Women’s Medical University, Tokyo, Japan; Nippon Medical School Graduate School of Medicine, JAPAN

## Abstract

Among antineutrophil cytoplasmic antibody (ANCA)-associated vasculitides (AAV), granulomatosis with polyangiitis (GPA) and proteinase 3-ANCA-positive AAV (PR3-AAV) are prevalent in European populations, while microscopic polyangiitis (MPA) and myeloperoxidase-ANCA-positive AAV (MPO-AAV) are predominant in the Japanese. We previously demonstrated association of DRB1*09:01-DQB1*03:*03* haplotype, a haplotype common in East Asians but rare in the European populations, with MPA/MPO-AAV, suggesting that a population difference in *HLA-class II* plays a role in the epidemiology of this disease. To gain further insights, we increased the sample size and performed an extended association study of *DRB1* and *DPB1* with AAV subsets in 468 Japanese patients with AAV classified according to the European Medicines Agency algorithm (MPA: 285, GPA: 92, eosinophilic GPA [EGPA]: 56, unclassifiable: 35) and 596 healthy controls. Among these patients, 377 were positive for MPO-ANCA and 62 for PR3-ANCA. The significance level was set at α = 3.3x10^-4^ by applying Bonferroni correction. The association of DRB1*09:*01* with MPO-AAV was confirmed (allele model, P = 2.1x10^-4^, odds ratio [OR] = 1.57). Protective association of DRB1*13:*02* was detected against MPO-AAV (allele model, P = 2.3x10^-5^, OR = 0.42) and MPA (dominant model, P = 2.7x10^-4^, OR = 0.43). A trend toward increased frequency of DPB1*04:*01*, the risk allele for GPA in European populations, was observed among Japanese patients with PR3-AAV when conditioned on DRB1*13:*02* (P_adjusted_ = 0.0021, OR_adjusted_ = 3.48). In contrast, the frequency of DPB1*04:*01* was decreased among Japanese patients with MPO-AAV, and this effect lost significance when conditioned on DRB1*13:*02* (P_adjusted_ = 0.16), suggesting that DRB1*13:*02* or other allele(s) in linkage disequilibrium may be responsible for the protection. The differential association of DPB1*04:*01* with PR3-AAV and MPO-AAV and difference in DPB1*04:*01* allele frequencies between populations supported the hypothesis that the *HLA-class II* population difference may account in part for these epidemiologic characteristics. Furthermore, taken together with our previous observations, the haplotype carrying DRB1*13:*02* was suggested to be a shared protective factor against multiple autoimmune diseases.

## Introduction

An epidemiologic difference is clearly observed in antineutrophil cytoplasmic antibody (ANCA) -associated vasculitides (AAV) between European and East Asian populations. In a population-based prospective study, microscopic polyangiitis (MPA) and myeloperoxidase (MPO) -ANCA-positive AAV (MPO-AAV) were the predominant subtypes (>80%) in Japan, while granulomatosis with polyangiitis (GPA) and proteinase 3 (PR3) -ANCA-positive AAV (PR3-AAV) were the predominant subtypes (>60%) in the UK [1]. Differences in the genetic backgrounds among populations may play a role in such epidemiologic differences.

Human leukocyte antigens (HLA) have been intensively investigated for association with susceptibility to a number of autoimmune diseases. With respect to AAV in the populations of European ancestry, candidate gene studies with relatively small sample sizes suggested an association of the *DR4-DQ7* haplotype with the predisposition to both GPA and MPA in the UK [2], of the *DR1-DQ1* haplotype with GPA in the UK [3], and of DRB1*04 with end-stage renal disease accompanied by GPA in Germany [4]. On the other hand, protective association of DRB1*13 has been detected for GPA in Germany [4] and in the Netherlands [5]. Subsequently, association of DPB1*04:*01* with GPA has been reported in a moderately powered study in Germany [6].

Recently, two large-scale genome-wide association studies (GWAS) on AAV in European [7] and North American [8] populations detected the strongest associations in the major histocompatibility complex (*MHC*) region. In the European study, GPA and PR3-AAV showed the strongest association in the *HLA-DP* region, while MPA and MPO-AAV demonstrated the strongest association in the *HLA-DQ* region [7], which is generally in strong linkage disequilibrium (LD) with the *HLA-DR* region. Additionally, in the North American study (in which only GPA was studied), the strongest association was observed in the *HLA-DP* region. Using imputation, the investigators demonstrated that DPB1*04 is a risk allele group for GPA [8], supporting the results of a previous study in Germany [6].

Due to the rarity of AAV, few studies have examined the genetics in Asian populations. Our group has been conducting a nationwide multicenter collaborative study of AAV in Japan. Thus far, we have reported the association of DRB1*09:*01* and DQB1*03:*03*, which are in strong LD, with MPA and MPO-AAV [9–11]. Curiously, while these risk haplotypes are among the most frequent *HLA-class II* haplotypes in East Asian populations, these haplotypes are very rare in populations of European or African ancestry (Allele*Frequencies in Worldwide Populations, http://www.allelefrequencies.net/) [12]. Thus, it is possible that ethnic differences in the genetic backgrounds may be related to the differences in the incidence of AAV subsets.

Another remarkable feature of AAV in Japan was recently reported. Unlike in European populations, approximately half of the patients who were classified into GPA according to the European Medicines Agency (EMEA) algorithm [13] were positive for MPO-ANCA [14]. Therefore, the comparison between Japanese and European populations of the genetic backgrounds of patients with GPA will be of interest. With respect to GPA in Japan, only one study with a very small sample size reported an association between GPA and *DR9*, and that study was performed before the EMEA algorithm was proposed [15]. Furthermore, 19.9% of Japanese AAV patients who were eligible for classification by the EMEA algorithm (based on ANCA positivity and symptoms and signs of AAV) nonetheless remained “unclassifiable” according to the EMEA algorithm [14]. Interestingly, 93.5% of these unclassifiable AAV patients were positive for MPO-ANCA, and 63.5% presented with interstitial lung disease (ILD) [14], suggesting that this group may constitute a unique subset in the AAV spectrum.

In the present study, we substantially increased the sample size of Japanese patients with AAV from those of our previous studies, and performed an extended association analysis on *DRB1* and *DPB1* with AAV in the Japanese population. Thus, the work described here allowed us to assess whether additional *DRB1* and *DPB1* predispositional or protective alleles exist for MPA and MPO-AAV, and whether *DRB1* and/or *DPB1* are associated with GPA and PR3-AAV. *HLA-DQA1* and *DQB1* were not genotyped in this study, because these loci are in strong LD with *DRB1*, making it highly difficult to distinguish associations of these alleles based on the current sample size. Our observations indicated that DRB1*13:*02* was associated with protection against MPA and MPO-AAV. In agreement with a previous study in European populations, association of DPB1*04:*01* with the predisposition to PR3-AAV, but not with MPO-AAV, was suggested in the Japanese population. Taken together with our previous observations (which demonstrated protective association of DRB1*13:*02* with rheumatoid arthritis (RA) [16] and systemic lupus erythematosus (SLE) [17]), the haplotype carrying DRB1*13:*02* was suggested to be a common protective factor against multiple autoimmune diseases.

## Materials and Methods

### Patients and controls

Four hundred and sixty-eight Japanese patients with AAV and 596 healthy controls, including the 116 patients with MPO-AAV and 265 controls reported in our previous study [11], were recruited at the institutes participating in the Research Committee on Intractable Vasculitides and in the Research Group on Progressive Renal Diseases, both organized by the Ministry of Health, Labour, and Welfare of Japan, research groups organized by Sagamihara Hospital, National Hospital Organization, and the Tokyo Medical and Dental University. Information on the patients and healthy individuals is shown in S1 Table. The patients were classified according to the EMEA algorithm [13]. Two hundred and eighty-five cases were classified as MPA, 92 as GPA, 56 as EGPA, and 35 were considered unclassifiable AAV. Among all patients, 377 and 62 were positive for MPO-ANCA and PR3-ANCA by ELISA, respectively.

### Ethics statement

This study was reviewed and approved by the Ethics Committees of the University of Tsukuba, National Hospital Organization Sagamihara Hospital, the Tokyo Medical and Dental University, Okayama University, Kyoto University, Kagawa University, Juntendo University, St. Marianna University, Kanazawa University, University of Tokyo, Kyorin University, Saitama Medical Center Hospital, the University of Miyazaki, Toho University, Kobe University Hospital, Kitano Hospital, Shimane University, Nagoya City University, Ehime University, Jichi Medical University, Kyoto Prefectural University, Tokyo Medical University Hachioji Medical Center, Kitasato University Hospital, Hamamatsu University, National Hospital Organization Shimoshizu National Hospital, Tenri Hospital, Tokyo Metropolitan Geriatric Hospital and Institute of Gerontology, Hyogo University, Kawasaki Municipal Hospital, Sendai Shakaihoken Hospital, Tokyo Women’s Medical College, Kyoundo Hospital, Tokyo Metropolitan Komagome Hospital, Ome Municipal General Hospital, Teikyo University, Hokkaido University, Fukuoka University, Okayama Saiseikai General Hospital, Aichi Medical University, Asahikawa Medical University, Kyushu University, Iwate Prefectural Central Hospital, and Nagasaki University. Written informed consent was obtained from all participants, except for some healthy individuals who donated blood (for use in medical genetics studies) before 2001, that is, prior to the implementation by the Japanese government of the Ethics Guidelines for Human Genome/Gene Analysis Research. From such healthy individuals, verbal informed consent for the genetics study had been obtained. In accordance with the Japanese Ethical Guidelines for Human Genome/Gene Analysis Research, such samples were anonymized in an unlinkable fashion, and were included in this study after review and approval by the Ethics Committee of the University of Tsukuba. This study was conducted according to the principles expressed in the Declaration of Helsinki.

### *HLA* allele typing

*HLA-DRB1* and *DPB1* genotypes were determined at the “four-digit” high-resolution level by polymerase chain reaction (PCR) -microtiter plate hybridization or PCR-sequence-specific oligonucleotide probes using WAKFlow HLA typing kits (Wakunaga Pharmaceutical Co., Ltd., Osaka, Japan). Amino acid sequences encoded by each *DRB1* or *DPB1* allele were based on the IMGT/HLA Database (http://www.ebi.ac.uk/ipd/imgt/hla/) [18].

### Statistical analysis

*DRB1* and *DPB1* alleles with frequencies ≥ 0.01 in healthy controls were examined for their association with AAV subsets by Fisher's exact test in 2 x 2 contingency tables. When one of the cell counts was zero, odds ratio (OR) and 95% confidence interval (CI) were calculated with Haldane’s modification by adding 0.5 to each cell count. Correction for multiple testing was done by Bonferroni correction. Statistical tests for 30 alleles (*DRB1*: 21, *DPB1*: 9) were carried out for each of five AAV subsets (MPA, GPA, EGPA, MPO-AAV, and PR3-AAV). Thus, the significance level was set at α = 3.3x10^-4^ (0.05/150). When a strong protective association with one allele is present in a multiallelic locus like *HLA*, the predispositional association of the other alleles tends to be overestimated, and the protective association of the other alleles tends to be underestimated. In order to confirm whether the apparent predispositional allele is really predispositional, and to detect additional predispositional or protective alleles, the relative predispositional effects (RPE) method was employed under the allele model, by consecutively excluding the most strongly associated allele from the next-round analysis, until no allele showed P_RPE_ < 0.05 [19]. Conditional logistic regression analysis was performed to detect the primary associated allele from the *DRB1* and *DPB1* alleles in LD. Association of each DRβ1 and DPβ1 amino acid variant with frequencies ≥ 0.01 in the healthy controls was tested by logistic regression analysis under the additive model. Statistical tests in 57 polymorphic amino acid positions (DRβ1: 38, DPβ1: 19) were conducted for each of two AAV subsets (MPO-AAV and PR3-AAV). Thus, in this analysis, the significance level was set at α = 4.4x10^-4^ (0.05/114) using Bonferroni correction. When three or more amino acid variants are present at the same position, only the P value for the comparison between the most strongly associated amino acid and all other amino acid variants is shown. Statistical power in this study, calculated with the PS (Power and Sample Size Calculation) program, is shown in S2 Table.

## Results

### HLA-DRB1

Association of *DRB1* alleles with AAV was tested using clinical disease classification according to the EMEA algorithm (Table 1 and S3 Table) as well as by ANCA specificity (Table 2 and S4 Table). This analysis permitted determination of the association of DRB1*09:*01* with AAV in a larger sample size than in our previous studies, along with assessment of the association of other predispositional or protective *DRB1* alleles with MPA and MPO-AAV. Predispositional association of the DRB1*09:*01* allele was confirmed in MPO-AAV (P = 2.1x10^-4^, OR = 1.57, 95%CI 1.24–1.98). In MPA, a trend for the association with this allele was detected (P = 7.7x10^-4^, OR = 1.56, 95%CI 1.21–2.01), although the association failed to achieve statistical significance after Bonferroni correction. Comparison of carrier frequency (dominant model) demonstrated essentially the same results (S3 and S4 Tables).

**Table 1:** *HLA-DRB1* allele frequencies in Japanese patients with MPA, EGPA, or GPA or in healthy controls.

*DRB1*	MPA (2n = 570)			EGPA (2n = 112)			GPA (2n = 184)			HC (2n = 1192)
	n (%)^a^	OR (95%CI)	P	n (%)^a^	OR (95%CI)	P	n (%)^a^	OR (95%CI)	P	n (%)^a^
01:01	41 (7.2)	1.24 (0.83–1.85)	0.30	8 (7.1)	1.23 (0.58–2.63)	0.53	14 (7.6)	1.32 (0.73–2.40)	0.41	70 (5.9)
04:01	6 (1.1)	0.74 (0.29–1.88)	0.66	0 (0.0)	0.30 (0.02–5.00)^b^	0.39	2 (1.1)	0.76 (0.17–3.31)	1.0	17 (1.4)
04:03	14 (2.5)	0.81 (0.43–1.51)	0.54	4 (3.6)	1.19 (0.42–3.40)	0.77	3 (1.6)	0.53 (0.16–1.75)	0.47	36 (3.0)
04:05	65 (11.4)	0.89 (0.65–1.21)	0.49	8 (7.1)	0.53 (0.25–1.11)	0.097	24 (13.0)	1.03 (0.65–1.64)	0.91	151 (12.7)
04:06	12 (2.1)	0.64 (0.33–1.22)	0.22	3 (2.7)	0.81 (0.25–2.68)	1.0	7 (3.8)	1.17 (0.51–2.65)	0.66	39 (3.3)
04:07	6 (1.1)	1.05 (0.39–2.80)	1.0	0 (0.0)	0.42 (0.02–7.14)^b^	0.61	2 (1.1)	1.08 (0.24–4.87)	1.0	12 (1.0)
04:10	9 (1.6)	1.11 (0.49–2.50)	0.83	3 (2.7)	1.90 (0.55–6.59)	0.24	4 (2.2)	1.54 (0.51–4.62)	0.51	17 (1.4)
08:02	21 (3.7)	1.34 (0.77–2.34)	0.30	1 (0.9)	0.32 (0.04–2.34)	0.35	13 (7.1)	2.67 (1.38–5.17)	0.0064	33 (2.8)
08:03	41 (7.2)	0.98 (0.67–1.45)	1.0	9 (8.0)	1.11 (0.54–2.27)	0.71	15 (8.2)	1.13 (0.64–2.00)	0.65	87 (7.3)
09:01	125 (21.9)	1.56 (1.21–2.01)	7.7E-04	24 (21.4)	1.51 (0.94–2.44)	0.10	35 (19.0)	1.30 (0.87–1.95)	0.19	182 (15.3)
11:01	16 (2.8)	1.61 (0.83–3.11)	0.16	3 (2.7)	1.53 (0.45–5.23)	0.45	5 (2.7)	1.56 (0.58–4.18)	0.38	21 (1.8)
12:01	17 (3.0)	0.73 (0.42–1.29)	0.34	4 (3.6)	0.88 (0.31–2.49)	1.0	2 (1.1)	0.26 (0.06–1.09)	0.054	48 (4.0)
12:02	8 (1.4)	0.72 (0.32–1.63)	0.56	2 (1.8)	0.92 (0.22–3.97)	1.0	1 (0.5)	0.28 (0.04–2.07)	0.24	23 (1.9)
13:02	25 (4.4)	0.47 (0.30–0.74)	6.3E-04	6 (5.4)	0.58 (0.25–1.35)	0.29	9 (4.9)	0.53 (0.26–1.06)	0.084	106 (8.9)
14:03	17 (3.0)	1.33 (0.72–2.45)	0.41	2 (1.8)	0.78 (0.18–3.34)	1.0	1 (0.5)	0.24 (0.03–1.75)	0.16	27 (2.3)
14:05	12 (2.1)	0.73 (0.38–1.43)	0.43	1 (0.9)	0.31 (0.04–2.26)	0.36	3 (1.6)	0.56 (0.17–1.86)	0.47	34 (2.9)
14:06	4 (0.7)	0.69 (0.22–2.16)	0.60	2 (1.8)	1.79 (0.40–8.09)	0.34	2 (1.1)	1.08 (0.24–4.87)	1.0	12 (1.0)
14:54	24 (4.2)	1.54 (0.90–2.64)	0.11	4 (3.6)	1.30 (0.45–3.74)	0.55	3 (1.6)	0.58 (0.18–1.92)	0.47	33 (2.8)
15:01	28 (4.9)	0.71 (0.46–1.10)	0.14	10 (8.9)	1.34 (0.68–2.67)	0.43	9 (4.9)	0.71 (0.35–1.43)	0.42	81 (6.8)
15:02	71 (12.5)	1.16 (0.85–1.58)	0.34	13 (11.6)	1.07 (0.59–1.97)	0.75	25 (13.6)	1.28 (0.81–2.03)	0.32	130 (10.9)
16:02	1 (0.2)	0.17 (0.02–1.33)	0.073	1 (0.9)	0.89 (0.11–6.88)	1.0	3 (1.6)	1.63 (0.46–5.83)	0.44	12 (1.0)

HC: healthy controls, OR: odds ratio, CI: confidence interval. P values were calculated by Fisher’s exact test. Significance level was set at α = 3.3x10^-4^ by applying Bonferroni correction.

^a^ n (%) indicates the number and percentage of each allele among the total number of alleles in each group (twice the number of individuals).

^b^ OR and 95% CI were calculated using Haldane’s method when one of the cell counts was zero.

**Table 2:** *HLA-DRB1* allele frequencies in Japanese patients with MPO-AAV or PR3-AAV or in healthy controls.

*DRB1*	MPO-AAV (2n = 754)				PR3-AAV (2n = 124)			HC (2n = 1192)
	n (%)^a^	OR (95%CI)	P	P_RPE_	n (%)^a^	OR (95%CI)	P	n (%)^a^
01:01	56 (7.4)	1.29 (0.89–1.85)	0.19	0.30	8 (6.5)	1.11 (0.52–2.35)	0.84	70 (5.9)
04:01	7 (0.9)	0.65 (0.27–1.57)	0.40	0.30	2 (1.6)	1.13 (0.26–4.96)	0.70	17 (1.4)
04:03	18 (2.4)	0.79 (0.44–1.39)	0.48	0.33	2 (1.6)	0.53 (0.13–2.21)	0.57	36 (3.0)
04:05	74 (9.8)	0.75 (0.56–1.01)	0.059	0.020	17 (13.7)	1.10 (0.64–1.88)	0.78	151 (12.7)
04:06	24 (3.2)	0.97 (0.58–1.63)	1.0	0.79	1 (0.8)	0.24 (0.03–1.76)	0.17	39 (3.3)
04:07	7 (0.9)	0.92 (0.36–2.35)	1.0	0.82	2 (1.6)	1.61 (0.36–7.29)	0.39	12 (1.0)
04:10	12 (1.6)	1.12 (0.53–2.35)	0.85	1.0	3 (2.4)	1.71 (0.50–5.93)	0.43	17 (1.4)
08:02	36 (4.8)	1.76 (1.09–2.85)	0.023	0.044	2 (1.6)	0.58 (0.14–2.43)	0.77	33 (2.8)
08:03	51 (6.8)	0.92 (0.64–1.32)	0.72	0.47	14 (11.3)	1.62 (0.89–2.94)	0.11	87 (7.3)
09:01	166 (22.0)	1.57 (1.24–1.98)	2.1E-04*	0.0012	23 (18.5)	1.26 (0.78–2.04)	0.36	182 (15.3)
11:01	19 (2.5)	1.44 (0.77–2.70)	0.26	0.33	5 (4.0)	2.34 (0.87–6.33)	0.090	21 (1.8)
12:01	20 (2.7)	0.65 (0.38–1.10)	0.13	0.077	3 (2.4)	0.59 (0.18–1.93)	0.62	48 (4.0)
12:02	6 (0.8)	0.41 (0.17–1.01)	0.054	0.035	2 (1.6)	0.83 (0.19–3.58)	1.0	23 (1.9)
13:02	30 (4.0)	0.42 (0.28–0.64)	2.3E-05*	-	9 (7.3)	0.80 (0.40–1.63)	0.62	106 (8.9)
14:03	21 (2.8)	1.24 (0.69–2.20)	0.55	0.65	0 (0.0)	0.17 (0.01–2.81)^b^	0.10	27 (2.3)
14:05	13 (1.7)	0.60 (0.31–1.14)	0.13	0.097	4 (3.2)	1.14 (0.40–3.25)	0.78	34 (2.9)
14:06	8 (1.1)	1.05 (0.43–2.59)	1.0	1.0	1 (0.8)	0.80 (0.10–6.20)	1.0	12 (1.0)
14:54	28 (3.7)	1.35 (0.81–2.26)	0.29	0.35	5 (4.0)	1.48 (0.57–3.85)	0.40	33 (2.8)
15:01	43 (5.7)	0.83 (0.57–1.21)	0.39	0.22	6 (4.8)	0.70 (0.30–1.63)	0.57	81 (6.8)
15:02	97 (12.9)	1.21 (0.91–1.60)	0.19	0.39	14 (11.3)	1.04 (0.58–1.87)	0.88	130 (10.9)
16:02	5 (0.7)	0.66 (0.23–1.87)	0.62	0.46	0 (0.0)	0.38 (0.02–6.44)^b^	0.62	12 (1.0)

HC: healthy controls, OR: odds ratio, CI: confidence interval. P values were calculated by Fisher’s exact test. RPE: relative predispositional effect. RPE analysis was performed by excluding DRB1*13:*02* allele from cases and controls. P values considered significant after Bonferroni correction (<3.3x10^-4^) are shown in bold with an asterisk.

^a^ n (%) indicates the number and percentage of each allele among the total number of alleles in each group (twice the number of individuals).

^b^ OR and 95% CI were calculated using Haldane’s method when one of the cell counts was zero.

In addition, significant protective association with DRB1*13:*02* was newly detected against MPO-AAV (allele model, P = 2.3x10^-5^, OR = 0.42, 95%CI 0.28–0.64, dominant model, P = 1.1x10^-5^, OR = 0.39, 95%CI 0.25–0.60) and MPA (dominant model, P = 2.7x10^-4^, OR = 0.43, 95%CI 0.26–0.68) (Table 2, S3 and S4 Tables). In order to exclude potential over- and underestimation caused by strongly associated alleles in a multiallelic locus such as *HLA*, the RPE method was employed. Thus, association of the other alleles was tested after excluding the most strongly associated DRB1*13:*02* allele from the patients and controls. In MPO-AAV, the tendency toward positive association of DRB1*09:*01* was still observed (P_RPE_ = 0.0012) (Table 2), suggesting that the predispositional association of DRB1*09:*01* with MPO-AAV was not a secondary phenomenon caused by protective association with DRB1*13:*02*.

With respect to other AAV subsets, a tendency toward positive association was detected between GPA and DRB1*08:*02* (P = 0.0064, OR = 2.67, 95%CI 1.38–5.17) (Table 1). Essentially identical results were observed in the analysis of carrier frequency (dominant model) (S3 and S4 Tables).

### HLA-DPB1

Next, association of *HLA-DPB1* with clinical classification (Table 3 and S5 Table) and with ANCA specificity (Table 4 and S6 Table) was examined. Protective association of DPB1*04:*01* with MPO-AAV was detected (allele frequency: P = 2.1x10^-4^, OR = 0.40, 95%CI 0.24–0.67, carrier frequency: P = 1.2x10^-4^, OR = 0.38, 95%CI 0.22–0.64) (Table 4 and S6 Table). Essentially the same results were observed in MPA (Table 3 and S5 Table). In RPE analysis, no significant association of the other alleles was detected in MPO-AAV (Table 4).

**Table 3:** *HLA-DPB1* allele frequencies in Japanese patients with MPA, EGPA, or GPA or in healthy controls.

*DPB1*	MPA (2n = 570)			EGPA (2n = 112)			GPA (2n = 184)			HC (2n = 1186)
	n (%)^a^	OR (95%CI)	P	n (%)^a^	OR (95%CI)	P	n (%)^a^	OR (95%CI)	P	n (%)^a^
02:01	155 (27.2)	1.19 (0.94–1.49)	0.14	23 (20.5)	0.82 (0.51–1.32)	0.49	50 (27.2)	1.19 (0.83–1.68)	0.36	284 (23.9)
02:02	24 (4.2)	1.33 (0.79–2.24)	0.33	4 (3.6)	1.12 (0.39–3.19)	0.78	4 (2.2)	0.67 (0.24–1.90)	0.64	38 (3.2)
03:01	29 (5.1)	1.36 (0.84–2.19)	0.21	4 (3.6)	0.94 (0.33–2.66)	1.0	6 (3.3)	0.85 (0.36–2.03)	0.84	45 (3.8)
04:01	19 (3.3)	0.51 (0.31–0.85)	0.0090	2 (1.8)	0.27 (0.07–1.11)	0.057	14 (7.6)	1.22 (0.67–2.21)	0.52	75 (6.3)
04:02	73 (12.8)	1.44 (1.05–1.97)	0.025	14 (12.5)	1.40 (0.77–2.53)	0.31	19 (10.3)	1.13 (0.67–1.88)	0.68	110 (9.3)
05:01	183 (32.1)	0.77 (0.62–0.95)	0.015	43 (38.4)	1.01 (0.68–1.51)	1.0	64 (34.8)	0.87 (0.63–1.20)	0.41	452 (38.1)
09:01	64 (11.2)	1.10 (0.80–1.52)	0.56	11 (9.8)	0.95 (0.50–1.82)	1.0	19 (10.3)	1.00 (0.60–1.67)	1.0	122 (10.3)
13:01	6 (1.1)	0.56 (0.23–1.40)	0.31	5 (4.5)	2.47 (0.92–6.66)	0.076	1 (0.5)	0.29 (0.04–2.16)	0.35	22 (1.9)
14:01	8 (1.4)	0.79 (0.35–1.79)	0.69	0 (0.0)	0.24 (0.01–4.00)^b^	0.25	5 (2.7)	1.55 (0.58–4.16)	0.38	21 (1.8)

HC: healthy controls, OR: odds ratio, CI: confidence interval, P values were calculated by Fisher’s exact test. Significance level was set at α = 3.3x10^-4^ by applying Bonferroni correction.

^a^ n (%) indicates the number and percentage of each allele among the total number of alleles in each group (twice the number of individuals).

^b^ OR and 95% CI were calculated using Haldane’s method when one of the cell counts was zero.

**Table 4:** *HLA-DPB1* allele frequencies in Japanese patients with MPO-AAV or PR3-AAV or in healthy controls.

*DPB1*	MPO-AAV (2n = 754)				PR3-AAV(2n = 124)			HC (2n = 1186)
	n^a^ (%)	OR (95%CI)	P	P_RPE_	n^a^ (%)	OR (95%CI)	P	n^a^ (%)
02:01	200 (26.5)	1.15 (0.93–1.41)	0.22	0.45	29 (23.4)	0.97 (0.63–1.50)	1.0	284 (23.9)
02:02	29 (3.8)	1.21 (0.74–1.98)	0.45	0.61	3 (2.4)	0.75 (0.23–2.46)	0.79	38 (3.2)
03:01	37 (4.9)	1.31 (0.84–2.04)	0.25	0.36	4 (3.2)	0.85 (0.30–2.39)	1.0	45 (3.8)
04:01	20 (2.7)	0.40 (0.24–0.67)	2.1E-04*	-	14 (11.3)	1.89 (1.03–3.45)	0.057	75 (6.3)
04:02	91 (12.1)	1.34 (1.00–1.80)	0.056	0.094	14 (11.3)	1.24 (0.69–2.25)	0.42	110 (9.3)
05:01	255 (33.8)	0.83 (0.69–1.00)	0.059	0.011	45 (36.3)	0.93 (0.63–1.36)	0.77	452 (38.1)
09:01	88 (11.7)	1.15 (0.86–1.54)	0.37	0.50	9 (7.3)	0.68 (0.34–1.38)	0.35	122 (10.3)
13:01	10 (1.3)	0.71 (0.33–1.51)	0.47	0.37	0 (0.0)	0.21 (0.01–3.45)^b^	0.26	22 (1.9)
14:01	11 (1.5)	0.82 (0.39–1.71)	0.72	0.59	3 (2.4)	1.38 (0.40–4.68)	0.49	21 (1.8)

HC: healthy controls, OR: odds ratio, CI: confidence interval. P values were calculated by Fisher’s exact test. RPE: relative predispositional effect. In MPO-AAV, RPE was performed by excluding DPB1*04:*01*. P value considered significant after Bonferroni correction (<3.3x10^-4^) is shown in bold with an asterisk.

^a^ n (%) indicates the number and percentage of each allele among the total number of alleles in each group (twice the number of individuals).

^b^ OR and 95% CI were calculated using Haldane’s method when one of the cell counts was zero.

When the association between *DPB1* and GPA/PR3-AAV was examined, DPB1*04:*01* (the risk allele for GPA in European populations) also showed a trend for association with predisposition to PR3-AAV in the Japanese population (P = 0.057, OR = 1.89, 95%CI: 1.03–3.45) (Table 4). An association with DPB1*04:*01* was not observed for GPA (P = 0.52) (Table 3).

### Conditional analysis of the DRB1*13:*02* and DPB1*04:*01* associations in AAV subsets

DRB1*13:*02* and DPB1*04:*01* alleles, which are both negatively associated with MPO-ANCA, were in moderate LD *(r*^*2*^ = 0.44). We therefore made an attempt to dissect these associations using logistic regression analysis (Table 5).

**Table 5:** Conditional logistic regression analysis between DRB1*13:*02* and DPB1*04:*01* in AAV subsets.

*HLA* allele	unconditioned		conditioned on DRB1*13:*02*		conditioned on DPB1*04:*01*	
	P	OR (95%CI)	P_adjusted_	OR_adjusted_ (95%CI)	P_adjusted_	OR_adjusted_ (95%CI)
MPA						
DRB1*13:*02*	0.0011	0.47 (0.30–0.73)	N/A	N/A	0.033	0.53 (0.29–0.93)
DPB1*04:*01*	0.011	0.51 (0.30–0.84)	0.57	0.83 (0.42–1.59)	N/A	N/A
GPA						
DRB1*13:*02*	0.076	0.53 (0.25–1.01)	N/A	N/A	0.0066	0.29 (0.11–0.68)
DPB1*04:*01*	0.52	1.21 (0.65–2.11)	0.015	2.55 (1.17–5.48)	N/A	N/A
MPO-AAV						
DRB1*13:*02*	6.6E-05	0.43 (0.28–0.64)	N/A	N/A	0.024	0.55 (0.32–0.91)
DPB1*04:*01*	4.4E-04	0.41 (0.24–0.66)	0.16	0.64 (0.33–1.18)	N/A	N/A
PR3-AAV						
DRB1*13:*02*	0.54	0.80 (0.37–1.54)	N/A	N/A	0.028	0.36 (0.14–0.86)
DPB1*04:*01*	0.042	1.86 (0.99–3.30)	0.0021	3.48 (1.55–7.78)	N/A	N/A

OR: odds ratio, CI: confidence interval, N/A: not applicable; P value, OR, and 95%CI and adjusted P value, OR, and 95%CI on the indicated *HLA* allele were calculated by logistic regression analysis under the additive model.

In MPO-AAV, the protective association of DPB1*04:*01* lost significance when conditioned on DRB1*13:*02* (unconditioned P = 4.4x10^-4^, P_adjusted_ = 0.16), while the tendency toward association of DRB1*13:*02* remained when conditioned on DPB1*04:*01* (unconditioned P = 6.6x10^-5^, P_adjusted_ = 0.024). These results suggested that the protective association of DPB1*04:*01* with MPO-AAV was secondary, and instead actually reflected LD with DRB1*13:*02*.

In contrast, although the association of DPB1*04:*01* was barely detectable in PR3-AAV (unconditioned P = 0.042), the tendency became apparent when conditioned on DRB1*13:*02* (P_adjusted_ = 0.0021). This observation suggested that DPB1*04:*01*, the risk allele for European GPA, also may contribute to susceptibility to PR3-AAV in the Japanese population. Similarly, DRB1*13:*02* showed a tendency toward association after conditioning on DPB1*04:*01* (P_adjusted_ = 0.028). The direction of association was opposite for DPB1*04:*01* (unconditioned OR = 1.86, OR_adjusted_ = 3.48) compared to DRB1*13:*02* (unconditioned OR = 0.54, OR_adjusted_ = 0.36). Thus, it is likely that the associations of DPB1*04:*01* and DRB1*13:*02* to PR3-AAV were attenuated by each other due to LD, and therefore were underestimated before conditioning. The same tendency also was observed in GPA after conditioning on the opposing allele. These results suggested that the protective effect of DRB1*13:*02* may be present across all subsets of AAV, while the predispositional effect of DPB1*04:*01* may be present in PR3-AAV and GPA.

### Effects of *DRB1* genotypes on MPO-AAV

Next, we calculated the OR for MPO-AAV with respect to *DRB1* genotypes. To do this, we classified each *DRB1* allele into three categories corresponding to DRB1*09:*01*, DRB1*13:*02*, and any remaining alleles (denoted by N for “neutral”). The results are shown in S1 Fig. When compared with genotype N/N as the referent, DRB1*09:*01/N* was associated with predisposition (OR = 1.65, P = 0.0013) and *13:*02/N* with protection (OR = 0.45, P = 0.0019). Significant association was not detected for *09:*01/09*:*01* or *13:*02/13*:*02*, presumably due to the small number of individuals homozygous for these alleles.

### Association of amino acid variations in DRβ1

Finally, we investigated the association of each amino acid variation in the DRβ1 and DPβ1 proteins with MPO-AAV and PR3-AAV using logistic regression analysis under the additive model. In DRβ1, 71E demonstrated the strongest association with the protection from MPO-AAV (P = 2.6x10^-5^, OR = 0.41) (S2A Fig). When conditioned on 71E, only 67F showed marginal predispositional association (P_adjusted_ = 4.9x10^-4^, OR = 1.46) (S2B Fig). When conditioned on both 71E and 67F, no further significant association was detected (S2C Fig).

With respect to DPβ1, significant association (P = 4.1x10^-4^, OR = 0.47) of 36A and 55A, which are in absolute LD, with MPO-ANCA was detected (S3A Fig). When conditioned on 36A, no further significant association was detected (S3B Fig).

Finally, because our observations indicated that the association of DPB1*04:*01* with MPO-AAV may be secondarily caused by DRB1*13:*02*, we examined the association of DPβ1 amino acids by conditioning on DRB1*13:*02*. With such conditioning, the association in DPβ1 amino acids was no longer significant (S3C Fig).

Significant association with PR3-ANCA was not detected for DRβ1 nor DPβ1 amino acids, probably due to the small sample size.

## Discussion

In this study, we performed a detailed analysis of the association of *HLA-DRB1* and *DPB1* using the “four-digit” high-resolution allele typing on 468 Japanese patients with AAV and 596 healthy controls. To our knowledge, this sample size is (to date) the largest among the AAV genetics studies reported for East Asian populations. In addition, the present work represents the first study to examine *DPB1* association with GPA in East Asian populations. In view of the remarkable differences in the epidemiology and genetic background between the European and East Asian populations [1], our study adds valuable information to the genetics research of AAV.

We confirmed the previously reported association of DRB1*09:*01* with MPO-AAV [9–11] in an extended association study. Moreover, this study identified DRB1*13:*02* as a protective allele for MPO-AAV and MPA. Although the protective effect of DRB1*13 has previously been reported for GPA or PR3-AAV in European populations [4,5], to our knowledge protective association with MPA or MPO-AAV has not previously been clearly demonstrated. This discrepancy likely reflects a lack of detection power due to the lower prevalence of MPO-AAV in the European populations.

Notably, we recently reported the protective association of DRB1*13:*02* against RA [16] and SLE [17]. Thus, in sharp contrast to DRB1*09:*01* (which also has been reported to be associated with predisposition to RA [20,21], SLE [21], type 1 diabetes [22], and myasthenia gravis [23]), DRB1*13:*02* or other allele(s) in strong LD appears to have a protective effect against multiple autoimmune rheumatic diseases.

A recent study in a Chinese population reported association of DRB1*11:*01* with susceptibility to MPA, but association of DRB1*09:*01* was not detected [24]. In the present study, although we detected a trend toward increased frequency of DRB1*11:*01* in some subgroups of AAV (Tables 1 and 2), this trend did not achieve statistical significance. We were unable to replicate association of DRB1*14:*54* with protection against overall AAV, nor DRB1*12:*02* association with predisposition to GPA in the Chinese [24]. The reasons for these discrepancies are not clear, although differences in *HLA* allele frequencies in the general population might play a role. In the Chinese study, the allele frequency of DRB1*11:*01* in the control group was 6.5% [24], which was considerably higher than the 1.8% allele frequency in the Japanese (Table 1).

In the *MHC* region, strong LD is detected. As for *DRB1*, strong LD is observed with *DQB1*. DRB1*09:*01* and DRB1*13:*02* were in nearly absolute LD with DQB1*03:*03* (r^2^ = 0.83) and DQB1*06:*04* (r^2^ = 0.89), respectively, in our healthy controls. This pattern makes it highly difficult to distinguish the association of the *DRB1* alleles from that of the *DQB1* alleles with the present sample size. Therefore, our observations only indicate that the major genetic factor for MPA/MPO-AAV is present in the *HLA-DR/DQ* region. This result is consistent with the finding from the European GWAS, which demonstrated the strongest association signal for MPA/MPO-AAV at a single nucleotide polymorphism (SNP) close to the *DQB1* locus (rs5000634) [7]. To fine-map the primary associated allele, the association study would need to be conducted using a larger sample size.

With regards to *DPB1*, the present study suggested the possibility that the DPB1*04:*01* allele may be associated with PR3-AAV in the Japanese population, as in Caucasian populations [6,8]. In contrast, DPB1*04:*01* was protective against MPO-AAV, although this protection was a secondary effect caused by LD with DRB1*13:*02*. In view of the striking population difference in the DPB1*04:*01* allele frequency in populations of Japanese (6.3%) (Table 3) and European extraction (e.g., 42.5% in USA) (Allele*Frequencies in Worldwide Populations http://www.allelefrequencies.net/) [12], DPB1*04:*01* may in part account for the epidemiologic difference in the prevalence of PR3-AAV. In fact, as already pointed out by Watts et al. [25], population allele frequency of DPB1*04:*01* is higher in northern Europe than in southern Europe (S4 Fig) based on Allele*Frequencies in Worldwide Populations http://www.allelefrequencies.net/) [12]. On the other hand, the population allele frequencies of DRB1*09:*01* and DQB1*03:*03* are low in both northern and southern Europe; no apparent north-south gradient is observed. Elucidation of whether the north-south gradient of *HLA* alleles can account for the geographic variation in incidence of MPA and MPO-AAV in Europe will require identification of *HLA* alleles that can explain the GWAS signal of rs5000634 [7] in European populations.

When the associations identified in the present work were analyzed with respect to the amino acid sequences, DRβ1 67F was associated with predisposition to, and 71E with protection against, MPO-AAV. Among the *DRB1* alleles having allele frequencies > 1% in the Japanese population, 67F is encoded by DRB1*08:*02*, *09:*01*, *11:*01*, and *12:*02*, while 71E is encoded only by DRB1*13:*02*. Among the alleles encoding 67F, the frequencies of all except for DRB1*12:*02* were nominally or significantly higher in patients with MPO-AAV (Table 2). Because amino acids at positions 67 and 71 influence antigenic peptide binding pockets P7 and P4 (respectively) [26], it is possible that the antigenic peptide motifs of these alleles may be involved in the molecular mechanism of association. On the other hand, the fact that DRB1*09:*01* and DRB1*13:*02* are associated with susceptibility to or protection against multiple autoimmune diseases with apparently no shared autoantigens suggests that a role is played by the non-*HLA* alleles carried by these *HLA* haplotypes (rather than the antigenic peptide specificity). These hypotheses are not mutually exclusive, and will require further investigation.

In the present study, associations generally appeared to be more prominent with ANCA specificity than with clinical classification (e.g., DPB1*04:*01* association with MPA: P = 0.0063, OR = 0.48; with MPO-AAV: P = 1.2x10^-4^, OR = 0.38; with GPA: P = 0.86, OR = 1.07; with PR3-AAV: P = 0.12, OR = 1.71), consistent with the results obtained by GWAS in European populations [7]. In this regard, it would be interesting to test the *HLA* association of the two potentially unique subsets in Japan, MPO-ANCA-positive GPA and MPO-ANCA-positive unclassifiable AAV [14], when sufficient numbers of samples become available. In the present study, both of these AAV subsets were included in MPO-ANCA-positive subset; even after excluding unclassifiable AAV from the analyses, the association of DRB1*09:*01* (P = 1.8x10^-4^) and DRB1*13:*02* (P = 1.4x10^-4^) with MPO-AAV remained significant.

One of the most interesting findings in our study is that the haplotype carrying DRB1*13:*02*, which we have reported to have protective association against RA [16] and SLE [17], is also protective against MPA and MPO-AAV, suggesting that this haplotype carries a common protective allele with shared efficacy against different autoimmune diseases. Identification (by fine mapping of the SNPs and deep sequencing) of the allele(s) responsible for the protection, and of these allele(s)’ molecular mechanism(s) would not only lead to better understanding of the etiopathogenesis of autoimmune diseases, but also to new molecular targets that could be effective for multiple autoimmune diseases.

There are several limitations in this study. Due to the rarity of AAV, this study is not an independent replication of our previous studies [9–11], but is instead an extension study. A replication study on a completely independent case-control set will be required to establish the *HLA* associations with AAV in the Japanese.

The sample sizes of GPA, EGPA, and PR3-AAV are especially small, due to the low prevalence of these subsets in the Japanese. Therefore, our results on these subsets suffer from low detection power (S2 Table), and significant associations may emerge when the sample size becomes larger, especially for the alleles which showed uncorrected P values < 0.05 but did not achieve significance following Bonferroni correction.

Another limitation is that we only focused on *DRB1* and *DPB1*, and did not examine *DQB1* (which is in strong LD with *DRB1*) or other *HLA* and non-*HLA* alleles. Therefore, at this point, we cannot exclude the possibility that the associations detected for *DRB1* and *DPB1* alleles may only tag other causal alleles. Such a possibility should be investigated in the future using high-density SNP mapping, imputation, and/or sequencing of the *MHC* region.

In conclusion, in addition to confirming the predispositional association of DRB1*09:*01* with MPO-AAV, this study identified the protective association of DRB1*13:*02* against MPA and MPO-AAV in the Japanese population. Taken together with our previous observations, DRB1*13:*02* itself (or another allele in LD with DRB1*13:*02*) was suggested to be a common protective factor against multiple autoimmune diseases.

## Supporting Information

S1 Fig Association of *HLA-DRB1* genotypes with MPO-AAV. Each *DRB1* allele was classified into DRB1*09:*01* (predispositional), DRB1*13:*02* (protective), or any of the remaining alleles (denoted by N for neutral). Odds ratio, 95% confidence interval, and P value of each genotype group were calculated against *N/N*. The numbers of patients and controls in each group are shown below.
(PPTX)

S2 Fig Association of DRβ1 amino acid variations with MPO-AAV. Association of each amino acid residue in the DRβ1 protein with MPO-AAV was analyzed using logistic regression analysis under the additive model. Amino acid residues with frequencies ≥ 0.01 in healthy controls were examined for their association. The significance level was set at α = 4.4x10^-4^ using Bonferroni correction. (A) Unconditioned P values (closed circles) are shown. When three or more amino acid variants are present at the same position, only the P value for the comparison between the most strongly associated amino acid and all other amino acid variants is shown. (B,C) P values conditioned on 71E (grey circles) and P values conditioned on 71E and 67F (open circles) were calculated for the amino acid variants shown in S2A Fig.
(PPTX)

S3 Fig Association of DPβ1 amino acid variations with MPO-AAV. Association of each amino acid residue in the DPβ1 protein with MPO-AAV was analyzed using logistic regression analysis under the additive model. Amino acid residues with frequencies ≥ 0.01 in healthy controls were examined for their association. The significance level was set at α = 4.4x10^-4^ using Bonferroni correction. (A) Unconditioned P values (closed circles) are shown. When three or more amino acid variants are present at the same position, only the P value for the comparison between the most strongly associated amino acid and all other amino acid variants is shown. (B,C) P values conditioned on 36A (grey circles) and P values conditioned on DRB1*13:*02* (open circles) were calculated for the amino acid variants shown in S3A Fig.
(PPTX)

S4 Fig North-south gradient of population DPB1*04:*01* allele frequency in Europeans. *DPB1* allele frequencies in European populations, aligned from north to south according to the latitude. The data were derived from Allele*Frequencies in Worldwide Populations (http://www.allelefrequencies.net/) [12].
(PPTX)

S1 Table EMEA classification and ANCA specificity of the subjects. SD: standard deviation, F: female, M: male, NA: not available.
(DOCX)

S2 Table Power calculation in the present study. NA: not available. Power calculation was conducted in each AAV subset and healthy controls (2n = 1192) using the PS (Power and Sample Size Calculation) program. Significance level was set at α = 3.3x10^-4^ (0.05/150).
(DOCX)

S3 Table *HLA-DRB1* allele carrier frequencies in Japanese patients with MPA, EGPA, or GPA or in healthy controls (dominant model). HC: healthy controls, OR: odds ratio, CI: confidence interval. P values were calculated by Fisher’s exact test. P values considered significant after Bonferroni correction (< 3.3x10^-4^) are shown in bold with an asterisk. ^a^n (%): number and percentage of individuals who carry the allele (either homozygotes or heterozygotes) among the total number of individuals in each group. ^b^OR and 95% CI were calculated using Haldane’s method when one of the cell counts was zero.
(DOCX)

S4 Table *HLA-DRB1* allele carrier frequencies in Japanese patients with MPO-AAV or PR3-AAV or in healthy controls (dominant model). HC: healthy controls, OR: odds ratio, CI: confidence interval. P values were calculated by Fisher’s exact test. P values considered significant after Bonferroni correction (< 3.3x10^-4^) are shown in bold with an asterisk. ^a^n (%): number and percentage of individuals who carry the allele (either homozygotes or heterozygotes) among the total number of individuals in each group. ^b^OR and 95% CI were calculated using Haldane’s method when one of the cell counts was zero.
(DOCX)

S5 Table *HLA-DPB1* allele carrier frequencies in Japanese patients with MPA, EGPA, or GPA or in healthy controls (dominant model). HC: healthy controls, OR: odds ratio, CI: confidence interval. P values were calculated by Fisher’s exact test. Significance level was set at α = 3.3x10^-4^ by applying Bonferroni correction. ^a^n (%): number and percentage of individuals who carry the allele (either homozygotes or heterozygotes) among the total number of individuals in each group. ^b^OR and 95% CI were calculated using Haldane’s method when one of the cell counts was zero.
(DOCX)

S6 Table *HLA-DPB1* allele carrier frequencies in Japanese patients with MPO-AAV or PR3-AAV or in healthy controls (dominant model). HC: healthy controls, OR: odds ratio, CI: confidence interval. P values were calculated by Fisher’s exact test. P values considered significant after Bonferroni correction (< 3.3x10^-4^) are shown in bold with an asterisk. ^a^n (%): number and percentage of individuals who carry the allele (either homozygotes or heterozygotes) among the total number of individuals in each group. ^b^OR and 95% CI were calculated using Haldane’s method when one of the cell counts was zero.
(DOCX)
