# Supplementary figures and images for: Protective Role of HLA-DRB1*13:02 against Microscopic Polyangiitis and MPO-ANCA-Positive Vasculitides in a Japanese Population: A Case-Control Study
Source: PLoS One. 2016 May 11;11(5):e0154393. doi: 10.1371/journal.pone.0154393 (PMC4868057; doi:10.1371/journal.pone.0154393)

## Slide 1
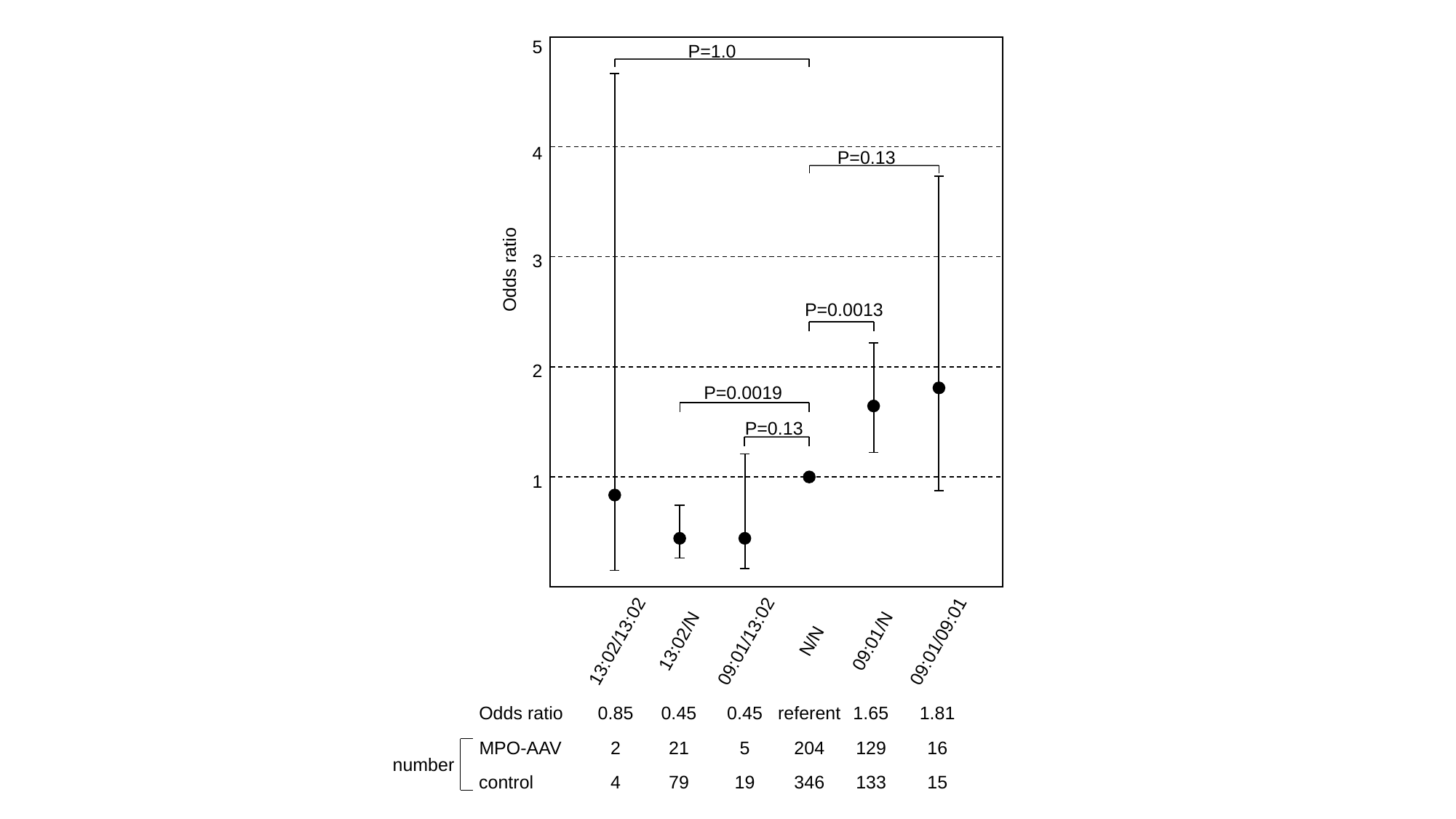

5
P=1.0
4
P=0.13
Odds ratio
3
P=0.0013
2
P=0.0019
P=0.13
1
13:02/13:02
09:01/13:02
09:01/09:01
13:02/N
09:01/N
N/N
Odds ratio
0.85
0.45
0.45
referent
1.65
1.81
MPO-AAV
2
21
5
204
129
16
number
control
4
79
19
346
133
15

Supplement: S1 Fig — Each DRB1 allele was classified into DRB1*09:01 (predispositional), DRB1*13:02 (protective), or any of the remaining alleles (denoted by N for neutral). Odds ratio, 95% confidence interval, and P value of each genotype group were calculated against N/N. The numbers of patients and controls in each group are shown below. (PPTX) [file pone.0154393.s001.pptx]
