# Supplementary material for: Protective Role of HLA-DRB1*13:02 against Microscopic Polyangiitis and MPO-ANCA-Positive Vasculitides in a Japanese Population: A Case-Control Study
Source: PLoS One. 2016 May 11;11(5):e0154393. doi: 10.1371/journal.pone.0154393 (PMC4868057; doi:10.1371/journal.pone.0154393)
Supplement: S2 Fig — Association of each amino acid residue in the DRβ1 protein with MPO-AAV was analyzed using logistic regression analysis under the additive model. Amino acid residues with frequencies ≥ 0.01 in healthy controls were examined for their association. The significance level was set at α = 4.4x10^-4^ using Bonferroni correction. (A) Unconditioned P values (closed circles) are shown. When three or more amino acid variants are present at the same position, only the P value for the comparison between the most strongly associated amino acid and all other amino acid variants is shown. (B,C) P values conditioned on 71E (grey circles) and P values conditioned on 71E and 67F (open circles) were calculated for the amino acid variants shown in S2A Fig. (PPTX) [file pone.0154393.s002.pptx]

## Slide 1
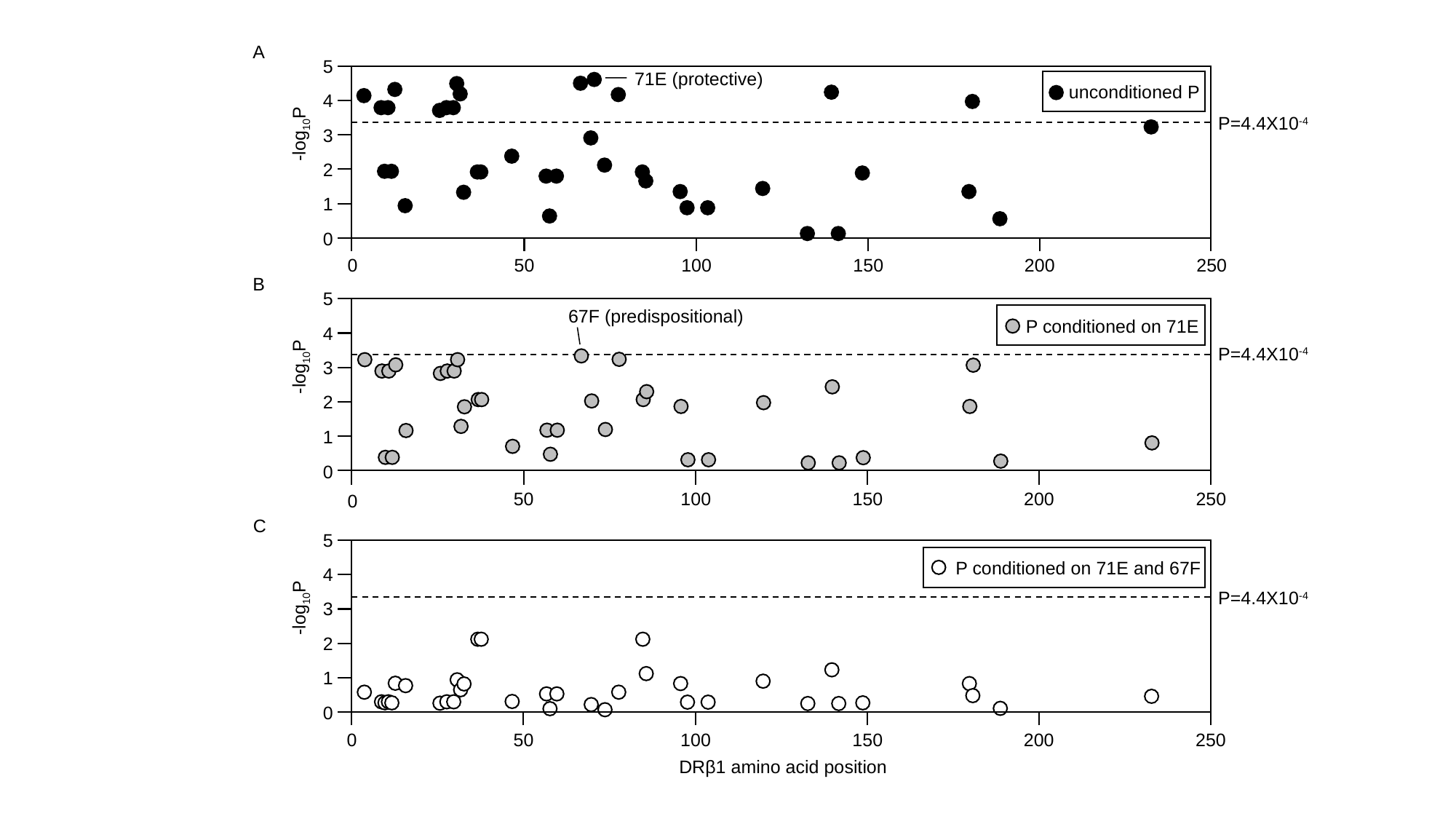

A
5
71E (protective)
unconditioned P
4
P=4.4X10-4
-log10P
3
2
1
0
0
50
100
150
200
250
B
5
67F (predispositional)
P conditioned on 71E
4
P=4.4X10-4
-log10P
3
2
1
0
50
100
150
200
250
0
C
5
P conditioned on 71E and 67F
4
P=4.4X10-4
-log10P
3
2
1
0
0
50
100
150
200
250
DRβ1 amino acid position
