# Supplementary material for: Protective Role of HLA-DRB1*13:02 against Microscopic Polyangiitis and MPO-ANCA-Positive Vasculitides in a Japanese Population: A Case-Control Study
Source: PLoS One. 2016 May 11;11(5):e0154393. doi: 10.1371/journal.pone.0154393 (PMC4868057; doi:10.1371/journal.pone.0154393)
Supplement: S4 Fig — DPB1 allele frequencies in European populations, aligned from north to south according to the latitude. The data were derived from Allele*Frequencies in Worldwide Populations (http://www.allelefrequencies.net/) [12]. (PPTX) [file pone.0154393.s004.pptx]

## Slide 1
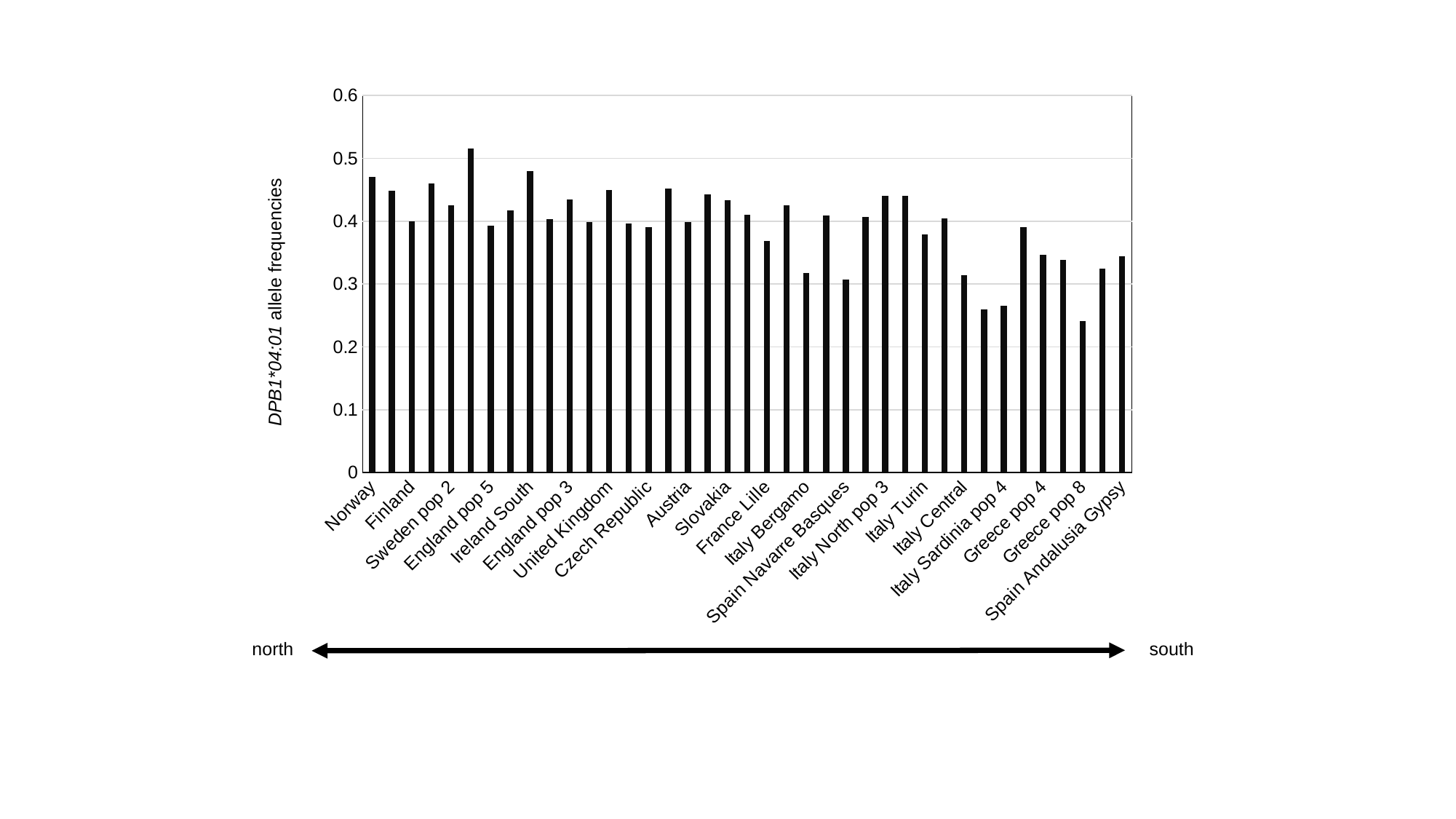

### Chart
| Category | |
|---|---|
| Norway | 0.47 |
| Sweden pop 3 | 0.448 |
| Finland | 0.4 |
| Sweden | 0.46 |
| Sweden pop 2 | 0.425 |
| Ireland Northern pop 3 | 0.516 |
| England pop 5 | 0.393 |
| England Norwest Mixed | 0.417 |
| Ireland South | 0.48 |
| Germany pop 2 | 0.403 |
| England pop 3 | 0.435 |
| Germany Essen | 0.398 |
| United Kingdom | 0.45 |
| Belgium | 0.396 |
| Czech Republic | 0.391 |
| France Rennes | 0.452 |
| Austria | 0.399 |
| Austria pop 2 | 0.443 |
| Slovakia | 0.433 |
| France West Breton | 0.41 |
| France Lille | 0.368 |
| France Southeasr | 0.425 |
| Italy Bergamo | 0.317 |
| France ceph | 0.409 |
| Spain Navarre Basques | 0.307 |
| Spain Gipuzkoa Basque | 0.407 |
| Italy North pop 3 | 0.44 |
| Italy North popn3 | 0.44 |
| Italy Turin | 0.379 |
| Spain Catalonia Girona | 0.404 |
| Italy Central | 0.314 |
| Italy Rome | 0.26 |
| Italy Sardinia pop 4 | 0.2653 |
| Greece | 0.39 |
| Greece pop 4 | 0.346 |
| Greece pop 6 | 0.3388 |
| Greece pop 8 | 0.241 |
| Spain Andalusia | 0.324 |
| Spain Andalusia Gypsy | 0.344 |DPB1*04:01 allele frequencies
north
south
