# Supplementary material for: Protective Role of HLA-DRB1*13:02 against Microscopic Polyangiitis and MPO-ANCA-Positive Vasculitides in a Japanese Population: A Case-Control Study
Source: PLoS One. 2016 May 11;11(5):e0154393. doi: 10.1371/journal.pone.0154393 (PMC4868057; doi:10.1371/journal.pone.0154393)
Supplement: S1 Table — SD: standard deviation, F: female, M: male, NA: not available. (DOCX) [file pone.0154393.s005.docx]

S1 Table. EMEA classification and ANCA specificity of the subjects.

|  | n | Age | Gender | MPO-ANCA positive | PR3-ANCA positive |
| --- | --- | --- | --- | --- | --- |
|  |  | (mean +/- SD) | (F/M ratio) | (n=377) | (n=62) |
| EMEA classification |  |  |  |  |  |
| MPA | 285 | 69.6+/-11.5 | 1.57 | 273 | 10 |
| GPA | 92 | 62.1+/-14.8 | 1.19 | 47 | 46 |
| EGPA | 56 | 57.5+/-15.3 | 1.55 | 28 | 4 |
| unclassifiable | 35 | 67.3+/-11.8 | 1.33 | 29 | 2 |
| Healthy controls | 596 | NA | 1.35 | NA | NA |

SD: standard deviation, F: female, M: male, NA: not available.
