# Supplementary material for: Protective Role of HLA-DRB1*13:02 against Microscopic Polyangiitis and MPO-ANCA-Positive Vasculitides in a Japanese Population: A Case-Control Study
Source: PLoS One. 2016 May 11;11(5):e0154393. doi: 10.1371/journal.pone.0154393 (PMC4868057; doi:10.1371/journal.pone.0154393)
Supplement: S2 Table — NA: not available. Power calculation was conducted in each AAV subset and healthy controls (2n = 1192) using the PS (Power and Sample Size Calculation) program. Significance level was set at α = 3.3x10^-4^ (0.05/150). (DOCX) [file pone.0154393.s006.docx]

S2 Table. Power calculation in this study.

|  |  | Power under the indicated odds ratio | | | | | |
| --- | --- | --- | --- | --- | --- | --- | --- |
| subset | Allele frequency | 0.4 | 0.6 | 0.8 | 1.2 | 1.4 | 1.6 |
| MPA (2n=570) | 0.01 | 0.001 | 0.000 | NA | 0.000 | 0.002 | 0.005 |
|  | 0.05 | 0.179 | 0.022 | 0.002 | 0.003 | 0.022 | 0.097 |
|  | 0.1 | 0.694 | 0.122 | 0.006 | 0.006 | 0.074 | 0.301 |
|  | 0.2 | 0.992 | 0.465 | 0.021 | 0.017 | 0.208 | 0.636 |
|  | 0.3 | 1.000 | 0.728 | 0.041 | 0.026 | 0.312 | 0.784 |
|  | 0.4 | 1.000 | 0.855 | 0.060 | 0.031 | 0.361 | 0.830 |
| GPA (2n=184) | 0.01 | NA | NA | NA | NA | NA | NA |
|  | 0.05 | 0.003 | 0.001 | NA | 0.001 | 0.007 | 0.028 |
|  | 0.1 | 0.052 | 0.007 | 0.001 | 0.002 | 0.018 | 0.075 |
|  | 0.2 | 0.425 | 0.049 | 0.003 | 0.004 | 0.042 | 0.171 |
|  | 0.3 | 0.783 | 0.132 | 0.006 | 0.006 | 0.059 | 0.231 |
|  | 0.4 | 0.928 | 0.226 | 0.009 | 0.006 | 0.063 | 0.246 |
| EGPA (2n=112) | 0.01 | NA | NA | NA | NA | NA | NA |
|  | 0.05 | NA | NA | NA | 0.001 | 0.004 | 0.015 |
|  | 0.1 | 0.007 | 0.002 | 0.000 | 0.001 | 0.010 | 0.037 |
|  | 0.2 | 0.117 | 0.013 | 0.001 | 0.002 | 0.019 | 0.077 |
|  | 0.3 | 0.372 | 0.040 | 0.002 | 0.003 | 0.025 | 0.099 |
|  | 0.4 | 0.615 | 0.078 | 0.004 | 0.003 | 0.025 | 0.100 |
| MPO-AAV (2n=754) | 0.01 | 0.004 | 0.001 | NA | 0.000 | 0.002 | 0.006 |
|  | 0.05 | 0.316 | 0.042 | 0.003 | 0.003 | 0.028 | 0.126 |
|  | 0.1 | 0.846 | 0.203 | 0.009 | 0.008 | 0.100 | 0.390 |
|  | 0.2 | 0.999 | 0.623 | 0.033 | 0.022 | 0.282 | 0.756 |
|  | 0.3 | 1.000 | 0.853 | 0.063 | 0.036 | 0.417 | 0.883 |
|  | 0.4 | 1.000 | 0.937 | 0.090 | 0.045 | 0.481 | 0.917 |
| PR3-AAV (2n=124) | 0.01 | NA | NA | NA | NA | NA | NA |
|  | 0.05 | 0.000 | NA | NA | 0.001 | 0.005 | 0.017 |
|  | 0.1 | 0.011 | 0.002 | 0.000 | 0.002 | 0.011 | 0.043 |
|  | 0.2 | 0.159 | 0.017 | 0.001 | 0.003 | 0.023 | 0.091 |
|  | 0.3 | 0.456 | 0.052 | 0.003 | 0.003 | 0.030 | 0.119 |
|  | 0.4 | 0.698 | 0.099 | 0.004 | 0.004 | 0.031 | 0.121 |

NA: not available. Power calculation was conducted in each AAV subset and healthy controls (2n=1192) using the PS (Power and Sample Size Calculation) program. Significance level was set at α=3.3x10^-4^ (0.05/150).
