# Supplementary material for: Protective Role of HLA-DRB1*13:02 against Microscopic Polyangiitis and MPO-ANCA-Positive Vasculitides in a Japanese Population: A Case-Control Study
Source: PLoS One. 2016 May 11;11(5):e0154393. doi: 10.1371/journal.pone.0154393 (PMC4868057; doi:10.1371/journal.pone.0154393)
Supplement: S3 Table — HC: healthy controls, OR: odds ratio, CI: confidence interval. P values were calculated by Fisher’s exact test. P values considered significant after Bonferroni correction (< 3.3x10^-4^) are shown in bold with an asterisk. an (%): number and percentage of individuals who carry the allele (either homozygotes or heterozygotes) among the total number of individuals in each group. bOR and 95% CI were calculated using Haldane’s method when one of the cell counts was zero. (DOCX) [file pone.0154393.s007.docx]

S3 Table. *HLA-DRB1* allele carrier frequencies in the Japanese patients with MPA, EGPA, GPA and healthy controls (dominant model).

| *DRB1* | MPA (n=285) | | |  | EGPA (n=56) | | |  | GPA (n=92) | | |  | HC (n=596) |
| --- | --- | --- | --- | --- | --- | --- | --- | --- | --- | --- | --- | --- | --- |
|  | n (%)^a^ | OR (95%CI) | P |  | n (%)^a^ | OR (95%CI) | P |  | n (%)^a^ | OR (95%CI) | P |  | n (%)^a^ |
| 01:01 | 40 (14.0) | 1.29 (0.85-1.96) | 0.27 |  | 8 (14.3) | 1.32 (0.60-2.90) | 0.51 |  | 14 (15.2) | 1.42 (0.76-2.64) | 0.30 |  | 67 (11.2) |
| 04:01 | 6 (2.1) | 0.78 (0.30-2.01) | 0.82 |  | 0 (0.0) | 0.31 (0.02-5.26)^b^ | 0.38 |  | 2 (2.2) | 0.81 (0.18-3.56) | 1.0 |  | 16 (2.7) |
| 04:03 | 13 (4.6) | 0.82 (0.42-1.57) | 0.63 |  | 4 (7.1) | 1.31 (0.45-3.85) | 0.55 |  | 3 (3.3) | 0.58 (0.17-1.91) | 0.46 |  | 33 (5.5) |
| 04:05 | 62 (21.8) | 0.90 (0.64-1.26) | 0.55 |  | 8 (14.3) | 0.54 (0.25-1.16) | 0.13 |  | 24 (26.1) | 1.14 (0.69-1.88) | 0.60 |  | 141 (23.7) |
| 04:06 | 12 (4.2) | 0.65 (0.33-1.26) | 0.22 |  | 3 (5.4) | 0.83 (0.25-2.78) | 1.0 |  | 7 (7.6) | 1.21 (0.52-2.80) | 0.65 |  | 38 (6.4) |
| 04:07 | 5 (1.8) | 0.87 (0.30-2.49) | 1.0 |  | 0 (0.0) | 0.41 (0.02-7.08)^b^ | 0.61 |  | 2 (2.2) | 1.08 (0.24-4.91) | 1.0 |  | 12 (2.0) |
| 04:10 | 9 (3.2) | 1.11 (0.49-2.52) | 0.83 |  | 3 (5.4) | 1.93 (0.55-6.79) | 0.24 |  | 4 (4.3) | 1.55 (0.51-4.71) | 0.51 |  | 17 (2.9) |
| 08:02 | 21 (7.4) | 1.36 (0.77-2.39) | 0.30 |  | 1 (1.8) | 0.31 (0.04-2.31) | 0.35 |  | 13 (14.1) | 2.81 (1.42-5.56) | 0.0055 |  | 33 (5.5) |
| 08:03 | 41 (14.4) | 1.05 (0.70-1.58) | 0.84 |  | 8 (14.3) | 1.04 (0.48-2.29) | 0.84 |  | 12 (13.0) | 0.94 (0.49-1.80) | 1.0 |  | 82 (13.8) |
| 09:01 | 113 (39.6) | 1.69 (1.25-2.27) | 6.6E-04 |  | 21 (37.5) | 1.54 (0.87-2.72) | 0.16 |  | 31 (33.7) | 1.31 (0.82-2.08) | 0.27 |  | 167 (28.0) |
| 11:01 | 16 (5.6) | 1.63 (0.84-3.17) | 0.15 |  | 3 (5.4) | 1.55 (0.45-5.37) | 0.45 |  | 5 (5.4) | 1.57 (0.58-4.28) | 0.38 |  | 21 (3.5) |
| 12:01 | 17 (6.0) | 0.74 (0.42-1.31) | 0.33 |  | 4 (7.1) | 0.90 (0.31-2.59) | 1.0 |  | 2 (2.2) | 0.26 (0.06-1.09) | 0.049 |  | 47 (7.9) |
| 12:02 | 8 (2.8) | 0.72 (0.32-1.63) | 0.56 |  | 2 (3.6) | 0.92 (0.21-4.02) | 1.0 |  | 1 (1.1) | 0.27 (0.04-2.05) | 0.23 |  | 23 (3.9) |
| 13:02 | 23 (8.1) | 0.43 (0.26-0.68) | **2.7E-04*** |  | 6 (10.7) | 0.58 (0.24-1.39) | 0.26 |  | 7 (7.6) | 0.40 (0.18-0.89) | 0.021 |  | 102 (17.1) |
| 14:03 | 17 (6.0) | 1.34 (0.72-2.49) | 0.41 |  | 2 (3.6) | 0.78 (0.18-3.37) | 1.0 |  | 1 (1.1) | 0.23 (0.03-1.73) | 0.16 |  | 27 (4.5) |
| 14:05 | 12 (4.2) | 0.75 (0.38-1.47) | 0.51 |  | 1 (1.8) | 0.31 (0.04-2.31) | 0.35 |  | 3 (3.3) | 0.58 (0.17-1.91) | 0.46 |  | 33 (5.5) |
| 14:06 | 4 (1.4) | 0.69 (0.22-2.17) | 0.60 |  | 2 (3.6) | 1.80 (0.39-8.26) | 0.34 |  | 2 (2.2) | 1.08 (0.24-4.91) | 1.0 |  | 12 (2.0) |
| 14:54 | 24 (8.4) | 1.62 (0.94-2.81) | 0.10 |  | 4 (7.1) | 1.36 (0.46-3.98) | 0.54 |  | 2 (2.2) | 0.39 (0.09-1.66) | 0.30 |  | 32 (5.4) |
| 15:01 | 28 (9.8) | 0.77 (0.49-1.22) | 0.31 |  | 10 (17.9) | 1.53 (0.74-3.17) | 0.29 |  | 9 (9.8) | 0.76 (0.37-1.59) | 0.61 |  | 74 (12.4) |
| 15:02 | 69 (24.2) | 1.22 (0.87-1.70) | 0.26 |  | 12 (21.4) | 1.04 (0.53-2.03) | 0.86 |  | 24 (26.1) | 1.34 (0.81-2.23) | 0.28 |  | 124 (20.8) |
| 16:02 | 1 (0.4) | 0.17 (0.02-1.32) | 0.072 |  | 1 (1.8) | 0.88 (0.11-6.93) | 1.0 |  | 3 (3.3) | 1.64 (0.45-5.93) | 0.44 |  | 12 (2.0) |

HC: healthy controls, OR: odds ratio, CI: confidence interval, P values were calculated by Fisher’s exact test. P value considered significant after Bonferroni correction (<3.3x10^-4^) is shown in bold with an asterisk. ^a^n (%): number and percentage of individuals who carry the allele (either homozygotes or heterozygotes) among the total number of individuals in each group. ^b^OR and 95% CI were calculated using Haldane’s method when one of the cell counts was zero.
