# Supplementary material for: Protective Role of HLA-DRB1*13:02 against Microscopic Polyangiitis and MPO-ANCA-Positive Vasculitides in a Japanese Population: A Case-Control Study
Source: PLoS One. 2016 May 11;11(5):e0154393. doi: 10.1371/journal.pone.0154393 (PMC4868057; doi:10.1371/journal.pone.0154393)
Supplement: S4 Table — HC: healthy controls, OR: odds ratio, CI: confidence interval. P values were calculated by Fisher’s exact test. P values considered significant after Bonferroni correction (< 3.3x10^-4^) are shown in bold with an asterisk. an (%): number and percentage of individuals who carry the allele (either homozygotes or heterozygotes) among the total number of individuals in each group. bOR and 95% CI were calculated using Haldane’s method when one of the cell counts was zero. (DOCX) [file pone.0154393.s008.docx]

S4 Table. *HLA-DRB1* allele carrier frequencies in the Japanese patients with MPO-AAV, PR3-AAV and healthy controls (dominant model).

| *DRB1* | MPO-AAV (n=377) | | |  | PR3-AAV (n=62) | | |  | HC (n=596) |
| --- | --- | --- | --- | --- | --- | --- | --- | --- | --- |
|  | n (%)^a^ | OR (95%CI) | P |  | n (%)^a^ | OR (95%CI) | P |  | n (%)^a^ |
| 01:01 | 55 (14.6) | 1.35 (0.92-1.98) | 0.14 |  | 8 (12.9) | 1.17 (0.53-2.56) | 0.68 |  | 67 (11.2) |
| 04:01 | 7 (1.9) | 0.69 (0.28-1.68) | 0.52 |  | 2 (3.2) | 1.21 (0.27-5.38) | 0.68 |  | 16 (2.7) |
| 04:03 | 17 (4.5) | 0.81 (0.44-1.47) | 0.55 |  | 2 (3.2) | 0.57 (0.13-2.43) | 0.76 |  | 33 (5.5) |
| 04:05 | 71 (18.8) | 0.75 (0.54-1.03) | 0.080 |  | 17 (27.4) | 1.22 (0.68-2.20) | 0.53 |  | 141 (23.7) |
| 04:06 | 24 (6.4) | 1.00 (0.59-1.69) | 1.0 |  | 1 (1.6) | 0.24 (0.03-1.78) | 0.16 |  | 38 (6.4) |
| 04:07 | 6 (1.6) | 0.79 (0.29-2.12) | 0.81 |  | 2 (3.2) | 1.62 (0.35-7.42) | 0.63 |  | 12 (2.0) |
| 04:10 | 12 (3.2) | 1.12 (0.53-2.37) | 0.85 |  | 3 (4.8) | 1.73 (0.49-6.08) | 0.42 |  | 17 (2.9) |
| 08:02 | 35 (9.3) | 1.75 (1.07-2.86) | 0.028 |  | 2 (3.2) | 0.57 (0.13-2.43) | 0.76 |  | 33 (5.5) |
| 08:03 | 50 (13.3) | 0.96 (0.66-1.40) | 0.85 |  | 12 (19.4) | 1.50 (0.77-2.95) | 0.25 |  | 82 (13.8) |
| 09:01 | 150 (39.8) | 1.70 (1.29-2.23) | **1.5E-04*** |  | 20 (32.3) | 1.22 (0.70-2.15) | 0.46 |  | 167 (28.0) |
| 11:01 | 19 (5.0) | 1.45 (0.77-2.74) | 0.25 |  | 5 (8.1) | 2.40 (0.87-6.61) | 0.088 |  | 21 (3.5) |
| 12:01 | 20 (5.3) | 0.65 (0.38-1.12) | 0.15 |  | 3 (4.8) | 0.59 (0.18-1.97) | 0.61 |  | 47 (7.9) |
| 12:02 | 6 (1.6) | 0.40 (0.16-1.00) | 0.052 |  | 2 (3.2) | 0.83 (0.19-3.61) | 1.0 |  | 23 (3.9) |
| 13:02 | 28 (7.4) | 0.39 (0.25-0.60) | **1.1E-05*** |  | 7 (11.3) | 0.62 (0.27-1.39) | 0.28 |  | 102 (17.1) |
| 14:03 | 21 (5.6) | 1.24 (0.69-2.23) | 0.54 |  | 0 (0.0) | 0.17 (0.01-2.75)^b^ | 0.099 |  | 27 (4.5) |
| 14:05 | 13 (3.4) | 0.61 (0.32-1.17) | 0.16 |  | 4 (6.5) | 1.18 (0.40-3.44) | 0.77 |  | 33 (5.5) |
| 14:06 | 8 (2.1) | 1.06 (0.43-2.61) | 1.0 |  | 1 (1.6) | 0.80 (0.10-6.24) | 1.0 |  | 12 (2.0) |
| 14:54 | 28 (7.4) | 1.41 (0.84-2.39) | 0.22 |  | 4 (6.5) | 1.22 (0.42-3.56) | 0.77 |  | 32 (5.4) |
| 15:01 | 42 (11.1) | 0.88 (0.59-1.32) | 0.61 |  | 6 (9.7) | 0.76 (0.31-1.82) | 0.68 |  | 74 (12.4) |
| 15:02 | 94 (24.9) | 1.26 (0.93-1.72) | 0.13 |  | 13 (21.0) | 1.01 (0.53-1.92) | 1.0 |  | 124 (20.8) |
| 16:02 | 5 (1.3) | 0.65 (0.23-1.87) | 0.62 |  | 0 (0.0) | 0.37 (0.02-6.39)^b^ | 0.62 |  | 12 (2.0) |

HC: healthy controls, OR: odds ratio, CI: confidence interval. P values were calculated by Fisher’s exact test. P values considered significant after Bonferroni correction (<3.3x10^-4^) are shown in bold with an asterisk. ^a^n (%): number and percentage of individuals who carry the allele (either homozygotes or heterozygotes) among the total number of individuals in each group. ^b^OR and 95% CI were calculated using Haldane’s method when one of the cell counts was zero.
