# Supplementary material for: Protective Role of HLA-DRB1*13:02 against Microscopic Polyangiitis and MPO-ANCA-Positive Vasculitides in a Japanese Population: A Case-Control Study
Source: PLoS One. 2016 May 11;11(5):e0154393. doi: 10.1371/journal.pone.0154393 (PMC4868057; doi:10.1371/journal.pone.0154393)
Supplement: S5 Table — HC: healthy controls, OR: odds ratio, CI: confidence interval. P values were calculated by Fisher’s exact test. Significance level was set at α = 3.3x10^-4^ by applying Bonferroni correction. an (%): number and percentage of individuals who carry the allele (either homozygotes or heterozygotes) among the total number of individuals in each group. bOR and 95% CI were calculated using Haldane’s method when one of the cell counts was zero. (DOCX) [file pone.0154393.s009.docx]

S5 Table. *HLA-DPB1* allele carrier frequencies in the Japanese patients with MPA, EGPA, GPA and healthy controls (dominant model).

| *DPB1* | MPA (n=285) | | |  | EGPA (n=56) | | |  | GPA (n=92) | | |  | HC (n=593) |
| --- | --- | --- | --- | --- | --- | --- | --- | --- | --- | --- | --- | --- | --- |
|  | n (%)^a^ | OR (95%CI) | P |  | n (%)^a^ | OR (95%CI) | P |  | n (%)^a^ | OR (95%CI) | P |  | n (%)^a^ |
| 02:01 | 139 (48.8) | 1.21 (0.91-1.61) | 0.19 |  | 21 (37.5) | 0.76 (0.43-1.34) | 0.40 |  | 44 (47.8) | 1.17 (0.75-1.81) | 0.50 |  | 261 (44.0) |
| 02:02 | 23 (8.1) | 1.32 (0.77-2.27) | 0.32 |  | 4 (7.1) | 1.16 (0.40-3.37) | 0.77 |  | 4 (4.3) | 0.68 (0.24-1.96) | 0.64 |  | 37 (6.2) |
| 03:01 | 29 (10.2) | 1.45 (0.88-2.37) | 0.15 |  | 4 (7.1) | 0.98 (0.34-2.85) | 1.00 |  | 6 (6.5) | 0.89 (0.37-2.16) | 1.0 |  | 43 (7.3) |
| 04:01 | 18 (6.3) | 0.48 (0.28-0.82) | 0.0063 |  | 2 (3.6) | 0.26 (0.06-1.11) | 0.049 |  | 12 (13.0) | 1.07 (0.56-2.06) | 0.86 |  | 73 (12.3) |
| 04:02 | 68 (23.9) | 1.41 (1.00-1.98) | 0.059 |  | 13 (23.2) | 1.36 (0.71-2.61) | 0.37 |  | 17 (18.5) | 1.02 (0.58-1.79) | 1.0 |  | 108 (18.2) |
| 05:01 | 154 (54.0) | 0.74 (0.56-0.98) | 0.040 |  | 36 (64.3) | 1.13 (0.64-2.00) | 0.77 |  | 46 (50.0) | 0.63 (0.40-0.98) | 0.040 |  | 364 (61.4) |
| 09:01 | 62 (21.8) | 1.18 (0.83-1.67) | 0.37 |  | 11 (19.6) | 1.04 (0.52-2.07) | 0.86 |  | 19 (20.7) | 1.11 (0.64-1.91) | 0.78 |  | 113 (19.1) |
| 13:01 | 6 (2.1) | 0.59 (0.23-1.47) | 0.30 |  | 5 (8.9) | 2.67 (0.97-7.38) | 0.064 |  | 1 (1.1) | 0.30 (0.04-2.25) | 0.34 |  | 21 (3.5) |
| 14:01 | 7 (2.5) | 0.69 (0.29-1.63) | 0.54 |  | 0 (0.0) | 0.24 (0.01-3.94)^b^ | 0.24 |  | 4 (4.3) | 1.24 (0.42-3.69) | 0.76 |  | 21 (3.5) |

HC: healthy controls, OR: odds ratio, CI: confidence interval. P values were calculated by Fisher’s exact test. Significance level was set at α=3.3x10^-4^ by applying Bonferroni correction. ^a^n (%): number and percentage of individuals who carry the allele (either homozygotes or heterozygotes) among the total number of individuals in each group. ^b^OR and 95% CI were calculated using Haldane’s method when one of the cell counts was zero.
