# Supplementary material for: Protective Role of HLA-DRB1*13:02 against Microscopic Polyangiitis and MPO-ANCA-Positive Vasculitides in a Japanese Population: A Case-Control Study
Source: PLoS One. 2016 May 11;11(5):e0154393. doi: 10.1371/journal.pone.0154393 (PMC4868057; doi:10.1371/journal.pone.0154393)
Supplement: S6 Table — HC: healthy controls, OR: odds ratio, CI: confidence interval. P values were calculated by Fisher’s exact test. P values considered significant after Bonferroni correction (< 3.3x10^-4^) are shown in bold with an asterisk. an (%): number and percentage of individuals who carry the allele (either homozygotes or heterozygotes) among the total number of individuals in each group. bOR and 95% CI were calculated using Haldane’s method when one of the cell counts was zero. (DOCX) [file pone.0154393.s010.docx]

S6 Table. *HLA-DPB1* allele carrier frequencies in the Japanese patients with MPO-AAV, PR3-AAV and healthy controls (dominant model).

| *DPB1* | MPO-AAV (n=377) | | |  | PR3-AAV(n=62) | | |  | HC (n=593) |
| --- | --- | --- | --- | --- | --- | --- | --- | --- | --- |
|  | n (%)^a^ | OR (95%CI) | P |  | n (%)^a^ | OR (95%CI) | P |  | n (%)^a^ |
| 02:01 | 180 (47.7) | 1.16 (0.90-1.51) | 0.26 |  | 25 (40.3) | 0.86 (0.50-1.46) | 0.59 |  | 261 (44.0) |
| 02:02 | 28 (7.4) | 1.21 (0.72-2.01) | 0.51 |  | 3 (4.8) | 0.76 (0.23-2.55) | 1.0 |  | 37 (6.2) |
| 03:01 | 37 (9.8) | 1.39 (0.88-2.20) | 0.19 |  | 4 (6.5) | 0.88 (0.31-2.55) | 1.0 |  | 43 (7.3) |
| 04:01 | 19 (5.0) | 0.38 (0.22-0.64) | **1.2E-04*** |  | 12 (19.4) | 1.71 (0.87-3.36) | 0.12 |  | 73 (12.3) |
| 04:02 | 84 (22.3) | 1.29 (0.93-1.77) | 0.14 |  | 13 (21.0) | 1.19 (0.62-2.27) | 0.61 |  | 108 (18.2) |
| 05:01 | 207 (54.9) | 0.77 (0.59-1.00) | 0.052 |  | 32 (51.6) | 0.67 (0.40-1.13) | 0.14 |  | 364 (61.4) |
| 09:01 | 86 (22.8) | 1.26 (0.92-1.72) | 0.17 |  | 9 (14.5) | 0.72 (0.35-1.51) | 0.49 |  | 113 (19.1) |
| 13:01 | 10 (2.7) | 0.74 (0.35-1.59) | 0.57 |  | 0 (0.0) | 0.21 (0.01-3.56)^b^ | 0.25 |  | 21 (3.5) |
| 14:01 | 9 (2.4) | 0.67 (0.30-1.47) | 0.35 |  | 3 (4.8) | 1.38 (0.40-4.78) | 0.49 |  | 21 (3.5) |

HC: healthy controls, OR: odds ratio, CI: confidence interval. P values were calculated by Fisher’s exact test. P value considered significant after Bonferroni correction (<3.3x10^-4^) is shown in bold with an asterisk. ^a^n (%): number and percentage of individuals who carry the allele (either homozygotes or heterozygotes) among the total number of individuals in each group. ^b^OR and 95% CI were calculated using Haldane’s method when one of the cell counts was zero.
